# Formulation and Optimization of Polymeric Nanoparticles for Intranasal Delivery of Lorazepam Using Box-Behnken Design: *In Vitro* and *In Vivo* Evaluation 

**DOI:** 10.1155/2014/156010

**Published:** 2014-07-14

**Authors:** Deepak Sharma, Dipika Maheshwari, Gilphy Philip, Ravish Rana, Shanu Bhatia, Manisha Singh, Reema Gabrani, Sanjeev K. Sharma, Javed Ali, Rakesh Kumar Sharma, Shweta Dang

**Affiliations:** ^1^Department of Biotechnology, Jaypee Institute of Information Technology, A-10 Sector 62, Noida, Uttar Pradesh 201307, India; ^2^Faculty of Pharmacy, Jamia Hamdard, Hamdard Nagar, New Delhi 110062, India; ^3^Division of CBRN Defense, Institute of Nuclear Medicine and Allied Sciences, Brig SK Mazumdar Marg, Delhi 110054, India

## Abstract

The aim of the present study was to optimize lorazepam loaded PLGA nanoparticles (Lzp-PLGA-NPs) by investigating the effect of process variables on the response using Box-Behnken design. Effect of four independent factors, that is, polymer, surfactant, drug, and aqueous/organic ratio, was studied on two dependent responses, that is, *z*-average and % drug entrapment. Lzp-PLGA-NPs were successfully developed by nanoprecipitation method using PLGA as polymer, poloxamer as surfactant and acetone as organic phase. NPs were characterized for particle size, zeta potential, % drug entrapment, drug release behavior, TEM, and cell viability. Lzp-PLGA-NPs were characterized for drug polymer interaction using FTIR. The developed NPs showed nearly spherical shape with *z*-average 167–318 d*·*nm, PDI below 0.441, and −18.4 mV zeta potential with maximum % drug entrapment of 90.1%. *In vitro* drug release behavior followed Korsmeyer-Peppas model and showed initial burst release of 21.7 ± 1.3% with prolonged drug release of 69.5 ± 0.8% from optimized NPs up to 24 h. *In vitro* drug release data was found in agreement with *ex vivo* permeation data through sheep nasal mucosa. *In vitro* cell viability study on Vero cell line confirmed the safety of optimized NPs. Optimized Lzp-PLGA-NPs were radiolabelled with Technitium-99m for scintigraphy imaging and biodistribution studies in Sprague-Dawley rats to establish nose-to-brain pathway.

## 1. Introduction

Lorazepam is the drug of choice for the treatment of status epilepticus but its poor aqueous solubility and side effects like shortness of breath, paralysis of hind legs, and loss of righting reflex limit its use in the therapy [1–3]. The parenteral formulations adopt polyethylene glycol 400, propylene glycol, and benzyl alcohol as cosolvents to improve the aqueous solubility [4] but are associated with injection site reactions and problem of precipitation of the drug on dilution [5]. Apart from the cosolvent approach, researchers have also explored the potential of emulsions [6] and cyclodextrins [7, 8] for improved parenteral delivery of lorazepam. However, both of these approaches have their own limitations. Emulsions have poor physical stability on long term storage, risk of emboli formation and require strict aseptic handling [9]. Whereas to produce parenteral formulation of lorazepam with desired properties, relatively high concentrations of cyclodextrin derivatives are required (15–30% w/v).

Different routes of administration have been used for delivery of benzodiazepines viz., oral tablets, buccal/sublingual formulations, rectal inserts, IV, and intranasal formulations. Oral tablets, rectal inserts have limitations such as first pass metabolism, slow onset of action, drug degradation, and low patient compliance. Buccal/sublingual formulations have also been reported as they bypass gastric and hepatic first pass metabolism; however, such formulations are often swallowed instead of being retained in the mouth cavity resulting in incomplete absorption through sublingual mucosa and delayed onset of action. The usage of these routes is further limited as administration requires patient cooperation, which is often not possible during an acute seizure [10].

Biodegradable nanoparticles have been extensively studied for nose-to-brain drug delivery, drug administered to the nasal cavity reaches to CNS through olfactory or trigeminal route [11, 12]. Patil et al. 2009 [13] reported the limitation of nasal mucosa as barrier and concluded that the drugs to be delivered could be either coadministered with absorption enhancers or encapsulated into an appropriate carrier system.

PLGA has been widely explored for preparation of polymeric nanoparticles and is well reported for mucoadhesive properties [14, 15], improved drug stability, and enhanced entrapment efficiencies [16–18].

Nanoprecipitation is a versatile method wherein an organic phase containing the polymer and drug is added to a dispersing phase which is a nonsolvent for the polymer but miscible with the diffusing solvent. The formation of particles happens spontaneously [19]. This method does not require extended shearing/stirring rates, sonication and is mostly suitable for compounds having a hydrophobic nature such as lorazepam, which is soluble in ethanol or acetone, but displays very limited solubility in water [20–22].

The size and surface charge of NPs play an important role in transport and absorption into the body. It has been reported in literature that the cellular uptake of negatively charged NPs is high because of strong and nonspecific interactions with the plasma membrane. Wilhelm et al. reported that negative charged particles get repelled by the negative charged domains of the membrane but they get adsorbed to the positive sites of the cell surface leading to local neutralization of the membrane and a subsequent bending of the membrane favoring in turn the formation of endocytosis invaginations [23].

In the case of NPs the particle size and distribution play important role. Huang and Donovan, 1996 studied transport of polystyrene nanoparticles (10–500 nm) across rabbit nasal respiratory epithelium and concluded that amine modified nanoparticles in the size range of up to 200 nm were transported via both paracellular and transcellular route [24].

It is hypothesized that PLGA nanoparticles based intranasal delivery of lorazepam besides providing for a noninvasive way of controlled drug delivery to brain [25, 26] would also be an efficient means of reducing the peripheral toxicity associated with lorazepam [14, 27]. PLGA NPs can entrap both water soluble and water insoluble drug molecules and prevent them from degradation and reduce drug toxicity. Drug release from NPs can follow both passive diffusion and slow matrix degradation which results in biphasic drug release mechanism with initial burst and thereafter controlled release [18].

In the present study lorazepam loaded PLGA NPs were formulated using nanoprecipitation approach and the effects of related process parameters were analyzed using Box-Behnken design. Particle size and the size distribution of the nanoparticles formulation is a critical parameter to be studied for intranasal delivery as this will decide the uptake of particles by nasal mucosa. Quality by design and process optimization is a recommended tool by regulatory agencies for product development. This kind of study will help in development of nanoparticles with desired properties and produce a reproducible and robust process for further scale up. Response surface methodology (RSM) is a well-known tool used for process optimization. Several designs are available under RSM such as central composite, Box-Behnken, and D-optimal design. In the present study Box-Behnken design was employed for process optimization as it generates fewer runs as compared to central composite design with 4 variables [28–31].

Cytotoxicity analysis of nanoparticles is vital to ensure that it does not present any risk to the patient or elicit an acute toxicity response [32, 33]. The toxicity of PLGA nanoparticles of lorazepam was studied using kidney epithelial cells extracted from African green monkey (Vero cell line, ATCC number CCL-81).

The formulation was further studied for* in vitro* and* ex vivo* drug release, scintigraphy, and biodistribution study in Sprague-Dawley rats.

## 2. Materials and Methods

### 2.1. Materials

Poly (D, L-lactide-co-glycolic acid) (PLGA) 50 : 50 (molecular weight 30,000–60,000) and poloxamer 407 were purchased from Sigma-Aldrich, St. Louis, USA. Lorazepam was purchased from R L Fine Chem., Bangalore, India. HPLC grade acetone and water were purchased from Fisher Scientific, Mumbai, India. All other solvents were of HPLC grade.

### 2.2. Experimental Design

Box-Behnken design was employed for constructing polynomial model for optimization of Lzp-PLGA-NPs keeping 4 independent and 2 dependent variables using Design Expert (version 8.0.0, Stat-Ease Inc., Minneapolis, Minnesota). Box-Behnken design was selected for the study as it generates fewer runs with 4 independent variables. The independent and dependent variables are listed in Table 1. The polynomial equation generated by the experimental design is as follows:

(1) Y=A0+A1X1+A2X2+A3X3  +A4X4+A5X1X2+A6X1X3+A7X1X4  +A8X2X3+A9X2X4+A10X3X4  +A11X12+A12X22+A13X32+A14X42,

where, *Y* is the measured response of the dependent variables, *A*_0_ is the intercept, *A*_1_ to *A*_14_ are the regression coefficients computed from the observed experimental values of *Y*. *X*_1_, *X*_2_, *X*_3_, and *X*_4_ are the coded value of the independent variables. *X*_*a*_ *X*_*b*_(*a*, *b* = 1,2, 3,4) and *X*_*i*_^2^ (*i* = 1,2, 3,4) represent the interaction and quadratic terms, respectively.

### 2.3. Nanoparticles Preparation

Lzp-PLGA-NPs were prepared using emulsion solvent evaporation (nanoprecipitation) method. During the process, the organic phase was prepared by dissolving accurately weighed PLGA and lorazepam in acetone as organic solvent. The organic phase was then added drop wise at the rate of 1ml/min into an aqueous phase containing surfactant (poloxamer 407) dissolved in water as aqueous solvent. The nanoparticles suspension was kept under continuous stirring at 300 rpm (RPM preoptimized, data not shown) for 3 h at 30°C to allow the complete evaporation of acetone, leaving behind the colloidal suspension of Lzp-PLGA-NPs in aqueous phase.

The colloidal nanosuspension was centrifuged at 12,000 rpm (Remi, Mumbai, India) for 30 min at 4°C to get the final nanoparticulate containing pellet as encapsulated lorazepam. The pellet was washed with deionized water twice to remove unentrapped drug from the surface of NPs. Nanoparticulate pellets were redispersed in water.

### 2.4. HPLC Method for Lorazepam

Reversed phase HPLC method was developed and validated as per USP monograph using HPLC isocratic system (Waters, Vienna, Austria) for analysis of lorazepam in prepared nanoparticles (USP Monograph Lorazepam, USP30-NF25, 2496). The instrumentation includes the stationary phase as nonpolar Sunfire column C-18 (250 ∗ 4.6 mm, 5 *μ*m) maintained at 30°C, mobile phase delivery system containing solvent reservoir and microprocessor controlled high pressure pump, sample injection device, and UV detector at *λ* max 230 nm. Filtered and degassed mixture of acetonitrile : water : glacial acetic acid (60 : 40 : 0.4) was used as mobile phase. The flow rate of mobile phase was maintained at 1 ml/min. Injection volume was kept at 20 *μ*L.

### 2.5. Drug Entrapment Efficiency and Percentage Drug Loading

Entrapment efficiency of lorazepam was calculated by determining the amount of free drug present in supernatant through HPLC method. The Lzp-PLGA-NPs suspension was centrifuged (Remi, Mumbai, India) at 12,000 rpm at 4°C for 30 min, washed twice with HPLC water and supernatant was collected. The amount of unentrapped drug was determined by the developed RP-HPLC method and the percentage drug entrapment and drug loading [34] of nanoparticles was calculated by using the following equations:

(2) Encapsulation  efficiency  (%)=((total  amount  of  the  drug−amount  of  the  free  drug)total  drug)    ×100

(3) Drug  Loading  (%) =((Amount  of  drug−Un  entrapped  drug)weight  of  Lzp-PLGA-NPs)  ×100.

### 2.6. Measurement of Particle Size

Average particle size (*z*-average) and polydispersity index (PDI) of the developed nanoparticles were determined by laser dynamic light scattering using Malvern Zetasizer (Malvern, Worcestershire, UK). Particle size investigation was performed in triplicate by diluting NPs suspension to 1/50 v/v in HPLC water.

The PDI value indicates the particle size distribution of nanoparticles in a given sample. Higher value of PDI indicates the distribution of NPs with variable size range which results in the formation of aggregates and could result in low stability of particle suspension and low homogeneity [35].

### 2.7. Zeta Potential

The nanoparticles suspension was diluted fifty times with HPLC water and zeta potential was measured using Malvern Zetasizer (Malvern, Worcestershire, UK). Zeta potential indicates the surface charge on the particles and was measured to determine the stability of nanoparticles in the suspension.

### 2.8. Transmission Electron Microscopy (TEM)

The morphology of formulation was observed under TEM (TECNAI 200 Kv TEM, Fei, Electron optics Oregon USA) by using negative staining method. A drop of NPs, diluted with water (1/50 times), was spread on a 200 mesh copper grid coated with carbon film and kept for about 3 min. A drop of phosphotungstic acid (2% w/w) was dripped on the grid for 30 sec and excess droplet was removed using a filter paper. Finally, the grid was air dried for about 2 h and then used for microscopic analysis.

### 2.9. Fourier Transform Infrared (FTIR) Analysis

FTIR analysis was performed to study the chemical interaction between drug and polymer using Perkin Elmer BX II (PerkinElmer, Massachusetts, USA). The samples were scanned in the IR range from 400 to 4000 cm^−1^.

### 2.10. *In Vitro *Drug Release Studies

*In vitro* release of lorazepam from Lzp-PLGA-NPs was evaluated by the dialysis bag diffusion technique [36–38]. Nanoparticles were prepared and centrifuged and drug entrapment was calculated. NPs pellet was redispersed in 2 mL methanolic PBS buffer solution (pH 6.4, 30% v/v methanol). Methanolic PBS was used, as lorazepam being poorly water soluble requires the use of media with surfactant or cosolvent to provide for the sink conditions. The redispersed pellet (5 mg/mL) was placed into the cellulose dialysis membrane (molecular weight cut-off 14000, Sigma-Aldrich, St. Louis USA) with average flat width of 25 mm in methanolic phosphate buffer pH 6.4 and tied to the paddle of dissolution apparatus (Veego, Delhi, India). Dissolution was done at 100 rpm and 37 ± 0.5°C. 2 mL sample was taken out from the dissolution vessel at 0, 15 min, 30 min, 1 h, 2 h, 4 h, 6 h, 8 h, 16 h, and 24 h duration and 2 mL fresh buffer was added subsequently every time to maintain sink conditions. Samples were analyzed using HPLC to calculate the drug released from the membrane into the buffer solution.

### 2.11. *Ex Vivo *Drug Release Behaviour

Du et al. reported that the morphology of the ovine mucosa is more comparable to that of humans because of the presence of ciliated and nonciliated cells, basal cells, goblet cells, and serous glands [39]. To correlate* in vitro* drug release behaviour of drug from Lzp-PLGA-NPs,* ex vivo* study on sheep nasal mucosa was performed using Franz diffusion cell. Sheep nasal mucosa was procured from slaughter house. Nasal mucosa was washed with phosphate buffer pH 6.4 and stored at −20°C. Nasal mucosa with a contact area of 1.53 cm^2^ was mounted on receptor compartment of the Franz diffusion cells (diameter 10 mm, 15 mL volume), with dermal face in contact with phosphate buffer (pH = 6.4). Two experimental sets in triplicates were performed keeping temperature 37 ± 0.5°C, 100 RPM, that is, optimized Lzp-PLGA-NPs and drug suspension. The formulation/drug suspension 5 mg/mL (NPs/drug resuspended in 2 mL phosphate buffer pH 6.4) was applied on the outer surface of the nasal mucosa. 2 mL of sample was withdrawn from receptor compartment at 0, 15 min, 30 min, 1 h, 2 h, 4 h, 6 h, 8 h, 16 h, and 24 h duration and replaced with 2 mL of fresh phosphate buffer to maintain sink conditions. Samples were analyzed using HPLC to calculate the drug released from the membrane into the buffer solution, and calculation was done accordingly in order to determine the diffusion kinetics.

### 2.12. Cell Viability Studies

The cytotoxicity analysis was carried out on Vero cell line (African green monkey kidney) by MTT assay to assess the cell viability by the tetrazolium intracellular reduction. Vero cell line was maintained in DMEM medium supplemented with 10% fetal bovine serum at 37°C in 5% CO_2_ atmosphere. The Vero cells were seeded at 1 × 10^5^ cells/mL and allowed to attach for 24 hours after which the cells were incubated with various concentration of plain lorazepam (LS), Lzp-PLGA-NPs, and corresponding placebo. The MTT assay depends on the cleavage of the yellow tetrazolium salt in to the purple formazan crystals by metabolic active cells. This cellular decline involves the pyridine nucleotide cofactors NADH and NADPH. The formazan crystals formed are solubilized by DMSO and consequential colored elucidation is quantified using a scanning multiwell spectrophotometer (ELISA reader). Values that are lower than the control cells indicate a reduction in the rate of cell proliferation. Conversely, a higher absorbance rate indicates an increase in cell proliferation.

### 2.13. Radiolabeling of Lorazepam Solution and Its Nanoparticles

Lorazepam (6 mg/mL) was radiolabeled using ^99m^Tc by direct labeling method using stannous chloride dihydrate solution (2 mg/mL in ethanol) as reducing agent. To the resultant mixture, 200 *μ*L of ^99m^Tc-pertechnetate (5-6 mci) was added gradually with continuous mixing. The mixture was incubated at room temperature for 30 min. The final volume was made up to 2 mL using 0.90% (w/v) sodium chloride (normal saline) solution. The radiolabeling efficiency was assessed using ascending instant thin layer chromatography. Silica gel coated fiber glass sheets (Gelman Sciences, Inc., Ann Arbor, MI USA) and solvent system consisting of acetone was used as mobile phases. The effect of incubation time and stannous chloride concentration on radiolabeling efficiency was studied to achieve optimum reaction conditions.

The optimized radiolabeled drug solution was used for development of Lzp-PLGA-NPs for scintigraphy and biodistribution study. Lorazepam suspension (^99m^Tc-LS) and lorazepam PLGA nanoparticles (^99m^Tc-Lzp-PLGA-NPs) were assessed for* in vitro* stability in normal saline solution and in rat plasma. The ^99m^Tc-LS and ^99m^Tc-Lzp-PLGA-NPs were further used to carry scintigraphic and biodistribution studies using nuclear medicine techniques.

### 2.14. Gamma Scintigraphy Imaging

Approval to carry out animal studies was obtained from the INMAS Institutional Animal Ethics Committee (IAEC), New Delhi, India, IAEC vide number INM/IAEC 2013/07/007 and their guidelines were followed throughout the study. The biodistribution and pharmacoscintigraphy studies were performed on Sprague-Dawley rats (male 2-3 months) weighing 180–200 g obtained from the Central Animal House Facility of INMAS, Delhi, India. All animals were given normal feed and filtered drinking water ad libitum. Rats were kept at normal room temperature of 25 ± 5°C.

Three rats for each formulation per time point were used in the study. 20 *μ*L of radiolabeled complex of ^99m^Tc-LS (5 mCi/mL) containing 0.04–0.050 mg lorazepam (equivalent to 0.2–0.25 mg/kg) was intravenously injected through the tail vein of the rat. Similarly, the 20 *μ*L of radiolabeled complex of ^99m^Tc-LS/Lzp-PLGA-NPs (5 mCi/mL) containing 0.040–0.050 mg lorazepam (equivalent to 0.2–0.25 mg/kg B.W.) was administered 10 *μ*L in each nostril.

The rats were held from the back in slanted position during nasal administration of formulations. The rats were anaesthetized using 0.4 mL ketamine hydrochloride intramuscular injection (50 mg/mL) and placed on the imaging platform. Imaging was performed using Single Photon Emission Computerized Tomography (SPECT, LC 75-005, Diacam, Siemens AG; Erlanger, Germany) gamma camera.

### 2.15. Biodistribution Studies

Three rats for each formulation per time point were used in the study. The radiolabeled complex of ^99m^Tc-LS (5 mCi/mL) containing 0.04–0.050 mg lorazepam (equivalent to 0.2–0.25 mg/kg) was injected through tail vein of rats. Similarly, the 20 *μ*L of radiolabeled complex of ^99m^Tc-LS/Lzp-PLGA-NPs (5 mCi/mL) containing 0.040–0.050 mg lorazepam (equivalent to 0.2–0.25 mg/kg B.W.) was administered (10 *μ*L) in each nostril. Prior to nasal administration of the formulations, the rats were anaesthetized using 0.4 mL ketamine hydrochloride intramuscular injection (50 mg/mL) and the formulations were instilled into the nostrils with the help of micropipette (20 *μ*L) attached with low density polyethylene tube having 0.1 mm internal diameter at the site of delivery. The rats were held from the back in slanted position during nasal administration of the formulations. The rats were sacrificed with mercy killing at predetermined time intervals and blood was collected through retro orbital vein. Subsequently, brain was extracted, washed twice using normal saline solution and made free from adhering tissue/fluid and weighed. The radioactivity present in blood and brain was measured using shielded well-type gamma scintillation counter.

The radiopharmaceutical uptake per gram in brain/blood was calculated as a fraction of administered dose. The results of radioactivity in different organs were recorded.

### 2.16. Accelerated Stability Studies

Optimized Lzp-PLGA-NPs were subjected to a stability testing for three months as per ICH guidelines at a temperature of 25° ± 2°C and 60% RH. Optimized Lzp-PLGA-NPs were analyzed for the change in *z*-average and percentage drug remaining.

### 2.17. Data Analysis

*In vitro* and* ex vivo* data are reported as mean ± SD (*n* = 3) and the difference between the groups was tested using two-way ANOVA, using Graph Pad Prism 5.0, and the interaction was found significant as *P* < 0.05.

## 3. Results and Discussion

Total 26 confirmatory runs with 2 centre points were developed by Box-Behnken design for optimization of polymeric NPs keeping 4 independent and 2 dependent variables. All developed NPs were subjected for characterization, that is, average particle size, polydispersity index, zeta potential, and percentage drug entrapment. The effect of independent variables on dependent variables was investigated and contour plots were developed (Table 2).

### 3.1. Zeta Potential Analysis

**Figure 1:**
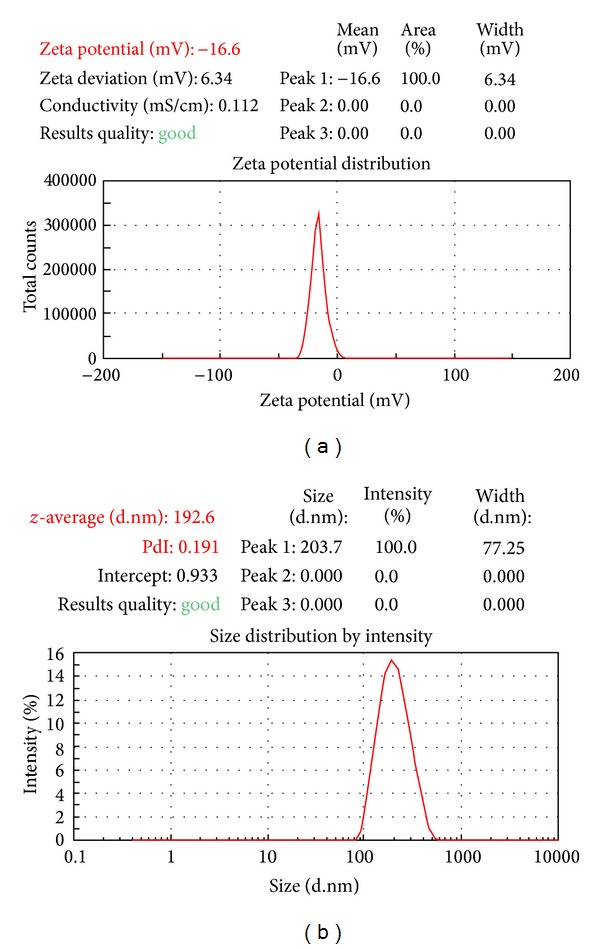
Zeta potential distribution and size distribution graph of drug loaded PLGA NPs (F 10).

Knowledge of the zeta potential for nanoparticles preparation could help to predict the fate of the nanoparticles* in vivo *and to assess the stability of colloidal systems. Surface charge on the particles could control the particles stability of the nanoparticulate formulation through strong electrostatic repulsion of particles with each other. In addition, from the zeta potential measurement, the dominated component on the particles surface was predicted as PLGA [40]. PLGA being negatively charged polymer imparts anionic nature to nanoparticles where zeta potential values were found in the range from −16.4 mV to −28.7 mV (Figure 1).

### 3.2. Effect of Independent Variables on *z*-Average

**Figure 2:**
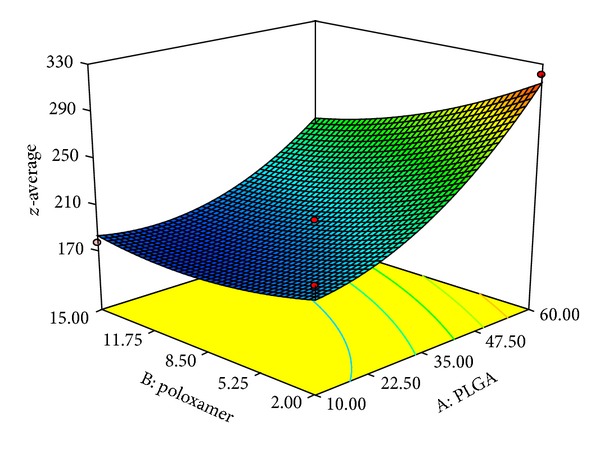
3D response surface plot showing effect of polymer (*X*_1_) and poloxamer concentration (*X*_2_) on *z*-average (*Y*_1_).

Polymer concentration is known to play an important role in controlling particle size along with release of drug from the matrix. *z*-Average of developed NPs was found in the range of 167 d*·*nm (F-20) −318 d*·*nm (F-12) for different variable combinations (Figure 2).

The effect on *z*-average can be explained by the following quadratic equation:

(4) Y1=197+44.08X1−21.38X2+7.67X3  +3.12X4−16.25X1X2+12.5X1X3  +−2.50X1X4−5.25X2X3+0.12X2X4−2.25X3X4  +24.94X12+11.0X22−0.44X32−6.13X42.

From the polynomial equation, a positive sign represented a synergistic effect, while a negative sign indicated an antagonistic effect. The model was found to be significant (*F*-value = 27.85; *P* < 0.0001). The values for predicted (0.9755) and adjusted (0.9493) *R*-square values were in reasonable agreement. The signal-to-noise ratio was found to be satisfactory as the observed adequate precision ratio of 19.9 is above 4. Thus, this model could be used to navigate the design space.

From the polynomial equation it is clear that factor *X*_1_, that is, PLGA, affected* z*-average of the polymeric NPs in the positive side and that the increase in concentration of factor *X*_1_ increases the *z*-average of NPs. The probable reason of increase in particle size could be that, during emulsification, increase in polymer concentration led to an increase in the viscosity of the organic phase which led to the formation of nanodroplets with larger size at interface at the stirring intensity (300 RPM). The effect of varying polymer concentration on particle size was found in agreement with Budhian et al. 2007 [41–43]. More viscous organic phase not only promotes the formation of larger size PLGA nanoparticles but also increases the amount of drug encapsulation inside nanoparticles [42, 44].

On the other side negative value of factor *X*_2_, that is, poloxamer, shows that the *z*-average of the NPs is indirectly proportional to the increasing poloxamer concentration. With decrease in poloxamer concentration the *z*-average of the NPs increases. Surfactants or stabilizers are usually involved in the process to modify the surface properties and to impart stability to nanoparticles. Surfactant allowed the formation of smaller droplet by increasing the interfacial stability of nanoparticles. With decrease in concentration of poloxamer, the mean diameter of PLGA nanoparticles was found to increase. The probable reason for formation of larger size nanoparticles could be reduced interfacial stability resulting from insufficient amount of surfactant leading to coalescence and aggregation of nanoparticles [42, 43].

**Figure 3:**
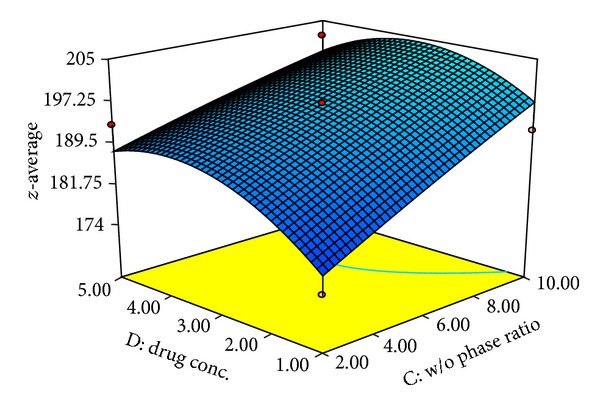
3D response surface plot shows effect of drug concentration and w/o phase ratio on *z*-average.

Factors *X*_3_ and *X*_4_, that is, w/o phase ratio and drug concentration, respectively, showed slight positive value as compared to factors *X*_1_ and *X*_2_ (Figure 3). Results showed that *z*-average is directly proportional to factors *X*_3_ and *X*_4_. Increasing drug concentration had no significant effect on the particle size of nanoparticles. However increasing w/o phase ratio from 2 : 1 to 10 : 1 is directly proportional to *z*-average. Increase in aqueous to organic phase ratio led to increase in particle size which could be due to small amount of organic phase volume available at the time of emulsification for lipophilic molecule lorazepam.

### 3.3. Effect on Percentage Drug Entrapment (*Y*_2_)

The percentage drug entrapment of developed NPs was found in the range of 65.5 (F-20)–90.1 (F-12).

The model proposed the following polynomial equation for effect of independent variables on percentage drug entrapment:

(5) Y2=86.65+3.76X1−2.10X2+0.39X3+8.53X4  −1.77X1X2−2X1X3−1.25X1X4−0.17X2X3  −0.15X2X4+0.25X3X4  −1.88X12−1.32X22−0.48X32−7.24X42.

The positive value before the factor indicates positive effect and negative value indicates negative effect on the percentage drug entrapment. The model was found to be significant (*F*-value = 24.9; *P* < 0.0001). The values for predicted (0.9279) and adjusted (0.9418) *R*-square values were in reasonable agreement. The signal-to-noise ratio was found to be satisfactory as the observed adequate precision ratio of 17.209 is above 4. Thus, this model could be used to navigate the design space.

**Figure 4:**
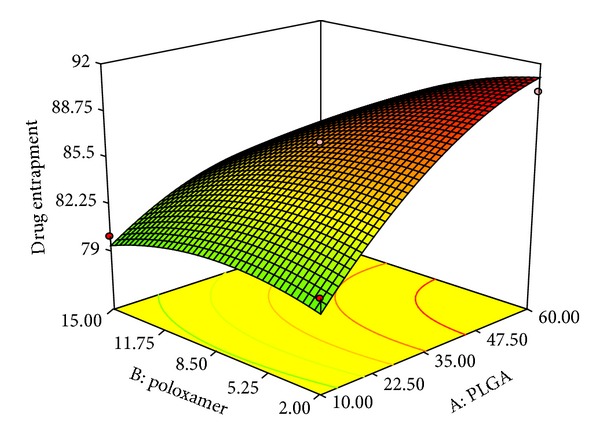
3D response surface plots showing effect of PLGA (*X*_1_) and poloxamer (*X*_2_) on % drug entrapment.

**Figure 5:**
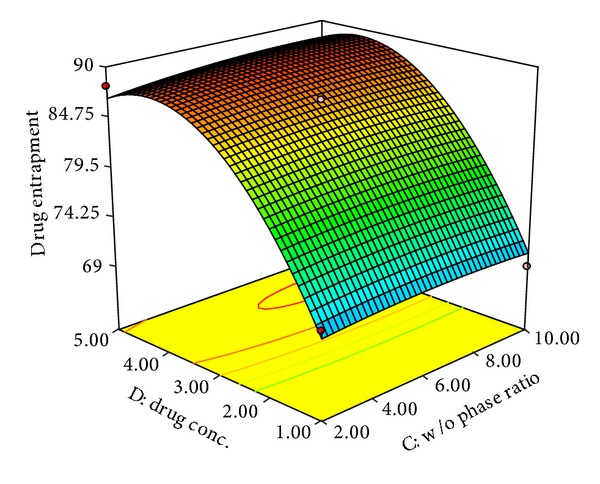
3D response surface plots showing effect of w/o phase ratio (*X*_3_) and drug concentration (*X*_4_) on % drug entrapment.

Analyzing polynomial equation it was found that percentage drug entrapment is increasing with increasing values of factors *X*_1_ (polymer concentration), *X*_3_ (w/o Phase ratio), and *X*_4_ (drug concentration), whereas factor *X*_2_ (surfactant) showed opposite effect. Factor *X*_3_ showed no significant effect on % drug entrapment (Figures 4 and 5).

The increased state of viscosity of organic phase due to increasing factor *X*_1_ could increase resistance to drug diffusion into the aqueous phase leading to the incorporation of more amount of drug inside NPs. Increased content of drug was found to be encapsulated in NPs with increase in particle size may be due to increase in length of diffusional pathway in aqueous phase (water) which reduces the drug loss and resulted in maximum encapsulation [45, 46].

The % drug entrapment efficiency in NPs is found to decrease with increasing in poloxamer concentration as indicated with negative coefficient value in polynomial equation. Lorazepam being a hydrophobic drug gets entrapped inside the PLGA nanoparticles formed at the interface and poloxamer stabilizes the nanoparticles by diffusing out the water molecules forming the polymer rich coacervate during the process of nanoprecipitation [47]. The increasing concentration of poloxamer may favor the higher aqueous solubility of drug by squeezing the nanoparticles which in turn increased the partition of drug inside aqueous phase and thereby resulted in decrease in entrapment of lorazepam inside the polymer. This finding was in agreement with Seju et al. 2011 [40, 44].

As the drug concentration or drug to polymer ratio increases the drug entrapment efficiency increased. The % drug entrapment in NPs is affected by drug-polymer interaction and drug miscibility in the organic solution. The importance of drug miscibility and drug-polymer interaction has been discussed by Panyam et al. 2004 for hydrophobic drug-polymer system of dexamethasone or flutamide-loaded PLGA/PLA nanoparticles [48]. Lorazepam being soluble in organic phase shows higher polymer interactions and miscibility with its increasing concentration and gets maximally entrapped inside the PLGA nanoparticles. Along with this, the hydrophobic nature of lorazepam enforces its maximum entrapment inside nanoparticles.

### 3.4. Data Analysis and Optimization

The optimum Lzp-PLGA-NPs formulation was selected by applying constraints on the dependent factors as shown in Table 1. Point prediction of the Design Expert software was used to determine the optimized NPs on the basis of closeness of desirability factor close to 1, which predicted the optimized process parameters to be *X*_1_ 10 mg/mL, *X*_2_ 9.42 mg/mL, *X*_3_ 10, and *X*_4_ 4.5 mg/mL with predicted values of responses *Y*_1_ 170.5 d*·*nm and *Y*_2_ 86.81%. The optimized formulation was developed and characterized for *z*-average and % drug entrapment. The experimental value for responses *Y*_1_ 168.2 d*·*nm with PDI 0.08 and *Y*_2_ 83.8% of optimized formulation was found in good agreement with the predicted values generated by the RSM and the result assures the validity of RSM model.

The percentage drug loading of optimized Lzp-PLGA-NPs was calculated using (3) and it was found to be 8.7%.

### 3.5. TEM Analysis

**Figure 6:**
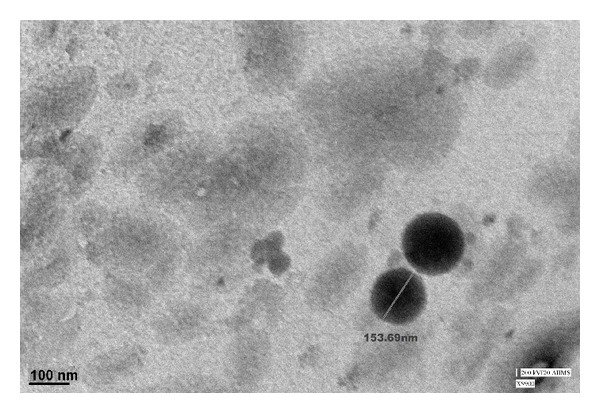
TEM images of the optimized Lzp-PLGA-NPs formulation.

TEM image (Figure 6) shows that the optimized formulation is nearly spherical in shape with particle size of 153.7 d*·*nm. Moreover, *z*-average gives the hydrodynamic size when the particles are suspended in aqueous media. TEM images would give a better understanding of the real geometric size of the particles and the correlation between process variables and particle size would be seen on a qualitative basis as well.

### 3.6. *In Vitro *Drug Release Studies

**Figure 7:**
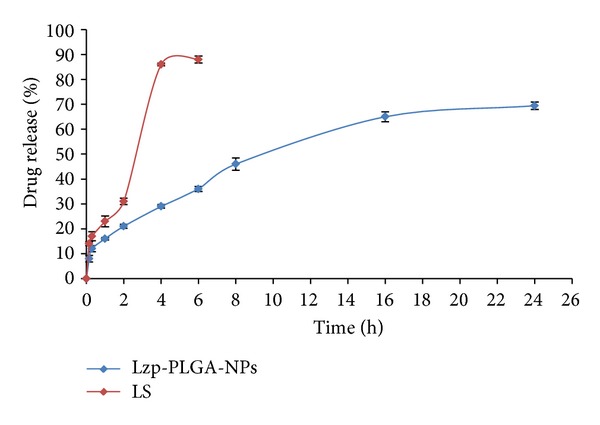
*In vitro* drug release profile from LS and optimized Lzp-PLGA-NPs.

The optimized Lzp-PLGA-NPs were subjected for* in vitro* drug release behaviour. Methanolic PBS was used as dissolution medium for evaluating the pattern of release of lorazepam from PLGA nanoparticles and plain aqueous drug suspension (LS) was used as control. The optimized Lzp-PLGA-NPs showed initial burst release of 21.7 ± 1.3%, whereas plain drug showed 31 ± 0.8% drug release (Figure 7). Thereafter optimized Lzp-PLGA-NPs showed sustained drug release with maximum drug release of 69.5 ± 0.8% in 24 h, while LS showed 86 ± 0.75% drug release within 4 h.

The drug release from Lzp-PLGA-NPs showed an initial burst release attributed to the drug release from drug associated near-particle surface which might have got desorbed upon contact with the dissolution medium [44, 49]. The particles of nanosize range led to a shorter average diffusion path for the matrix-entrapped drug molecules, which also causes faster diffusion. Thereafter, the release rate decreased which reflects not only the control of release rate of drug by the diffusion rate of the drug across the polymer matrix but also the issue of drug degradation after 24 hours of loading. Thus, it was clear that incorporation of lorazepam in PLGA nanoparticles could significantly sustain the release of lorazepam. The* in vitro* drug release data was analyzed using zero order, first order, Higuchi, and Korsmeyer-Peppas models. The graph for Korsmeyer-Peppas model was plotted between log time and log percentage drug remaining and the correlation coefficient was found (*r* ^2^) 0.947 for* in vitro* drug release, that is, almost unity for lorazepam NP and release exponent value (*n*) 0.460; therefore the best fit model for nanoparticles was Korsmeyer-Peppas model. The release exponent value (*n*) was below 0.5, which suggests lorazepam release from nanoparticles followed Fickian diffusion.

### 3.7. *Ex Vivo* Drug Release from NPs

**Figure 8:**
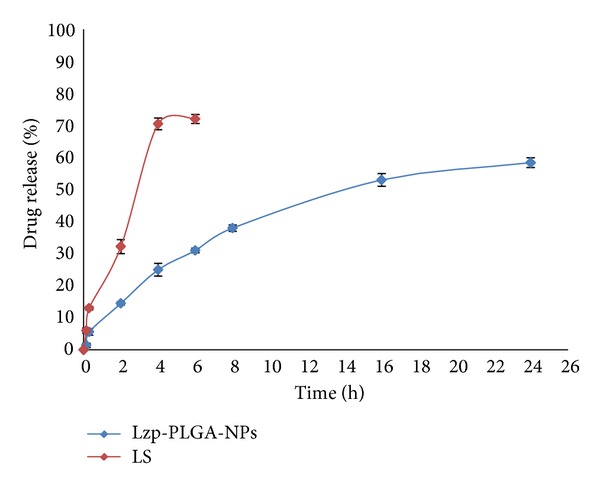
*Ex vivo *drug release profile from LS and optimized Lzp-PLGA-NPs through sheep nasal mucosa.

It is important to correlate* in vitro* drug release data with drug release behaviour through natural membrane. Sheep nasal mucosa was used as natural membrane and drug permeation was studied using Franz diffusion cell. The results showed that the* in vitro* data correlate with results of* ex vivo* drug permeation. Aqueous drug suspension (LS) was used as control for the study. Drug permeation from optimized Lzp-PLGA-NPs showed initial burst release with 14.5% within 2 h, whereas drug alone showed 32.2% drug permeation across nasal mucosa. Thereafter, drug release from optimized Lzp-PLGA-NPs showed controlled release maximum release of 58% up to 24 h, whereas LS showed maximum drug permeation of 71.5% within 4 h of study (Figure 8). The graph for Korsmeyer-Peppas model was plotted between log time and log percentage drug remaining and the correlation coefficient was found (*r* ^2^) 0.891 for* ex vivo* drug release, that is, almost unity for lorazepam NP and release exponent value (*n*) 0.43; therefore the best fit model for nanoparticles was Korsmeyer-Peppas model. The release exponent value (*n*) was below 0.5, which suggests lorazepam release from nanoparticles followed Fickian diffusion.

### 3.8. Fourier Transform Infrared Spectroscopy (FTIR)

**Figure 9:**
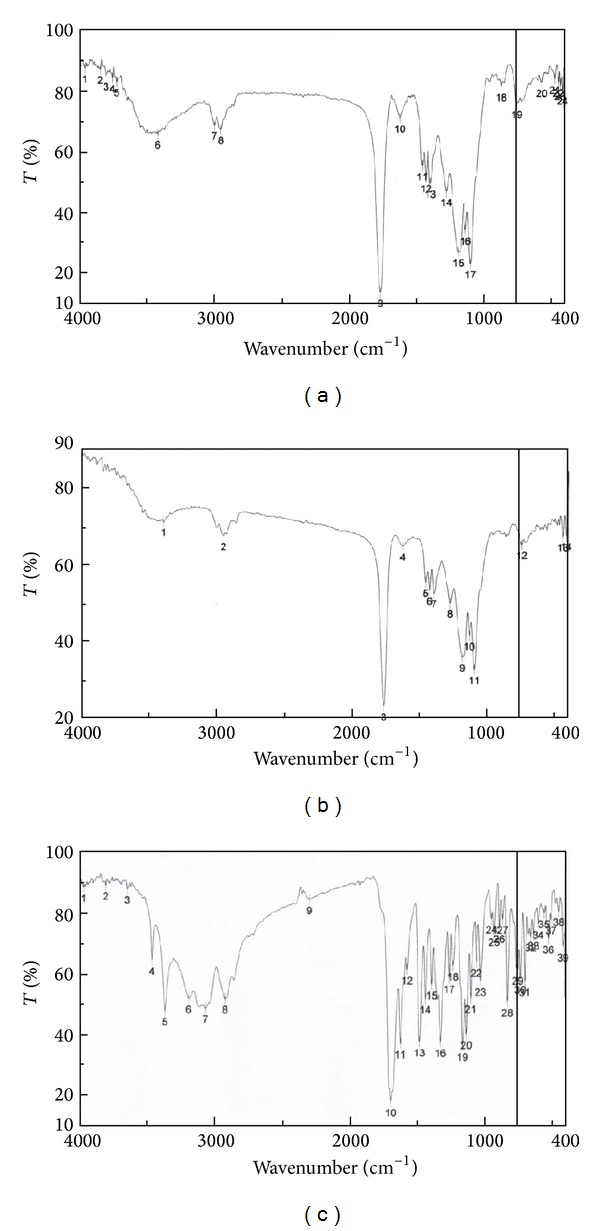
FTIR spectra of Lzp-PLGA-NPs (a), placebo (b), and pure lorazepam (c).

FTIR analysis of pure lorazepam, optimized Lzp-PLGA-NPs, and placebo-NPs was performed to investigate the interaction between drug and polymer.  Figure 9 shows FTIR spectra of lorazepam, Lzp-PLGA-NPs, and placebo. The pure lorazepam showed characteristic peaks of C–H alkanes stretch (2917 cm^−1^), aromatic rings (3186 cm^−1^, 3060 cm^−1^), O–H (3643 cm^−1^), C=O (1687 cm^−1^), N–H amines stretch (3459 cm^−1^, 3362 cm^−1^), and C–N amines stretch (1020 cm^−1^). The FTIR spectra of placebo showed some significant peaks due to the presence of PLGA: stretching –OH stretching (3290 cm^−1^), –CH, –CH_2_, –CH_3_ (2946 cm^−1^), carbonyl –C=O stretching (1759 cm^−1^), and C–O stretching (1093 cm^−1^). The FTIR spectra of Lzp-PLGA-NPs showed characteristic peaks of both PLGA and lorazepam that suggests no significant molecular interaction between drug and polymer [50, 51].

### 3.9. Cell Viability Analysis

**Figure 10:**
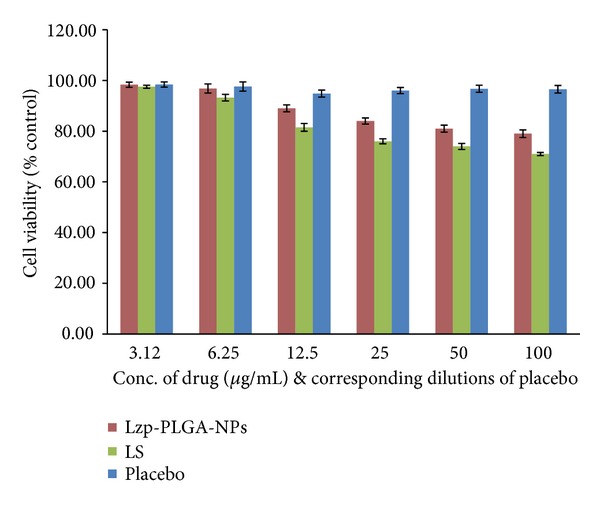
Vero cell viability analysis of Lzp-PLGA-NPs, LS, and placebo after 24 h via MTT assay. Error bar represents S.E, where *n* = 3.

Percentage cell viability of Lzp-PLGA-NPs, LS, and corresponding placebo was assessed on Vero cell line through MTT assay [44, 52]. Lzp-PLGA-NPs formulation and drug solution were studied in the concentration range from 3.125 *μ*g/mL to 100 *μ*g/mL and corresponding dilutions of placebo were checked for percentage cell viability for 24 h (Figure 10). Dose dependent cytotoxicity was observed with increase in concentration. Furthermore, Lzp-PLGA-NPs (89 ± 1.7%) and placebo (94.7% ± 1.5%) showed higher cell viability than the drug solution (81.5 ± 2.1%) at 12.5 *μ*g/mL concentration, indicating suitability of PLGA nanoparticles as carrier for lorazepam.

### 3.10. Gamma Scintigraphy Studies

**Figure 11:**
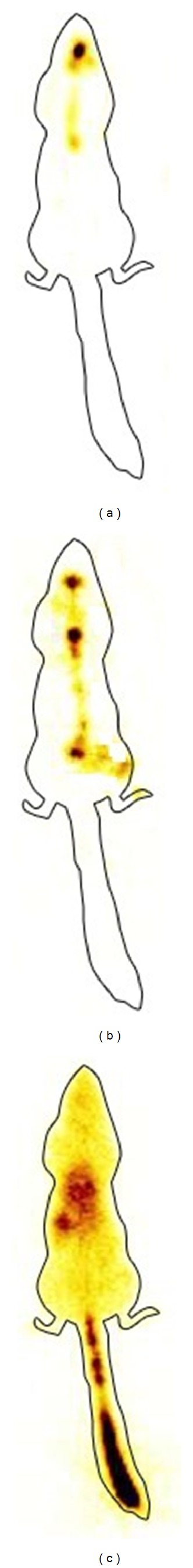
Gamma scintigraphy images of anterior view (from left to right) of rat at 2 h time point after i.n. administration of ^99m^Tc-Lzp-PLGA-NPs (a), i.n. administration of ^99m^Tc-LS (b), and i.v. administration of ^99m^Tc-LS (c).

LS and Lzp-PLGA-NPs formulations were effectively radiolabeled with Technetium-99m (^99m^Tc) and optimized for maximum labeling efficiency and stability. Radiolabelling efficiency was found to be 98.35% and 94.21% for LS and Lzp-PLGA-NPs, respectively. The optimal SnCl_2_ *·*2H_2_O concentration was found to be 2 mg/mL with an incubation time of 30 min. ^99m^Tc-LS/Lzp-PLGA-NPs were found to be stable in normal saline solution and in rat serum up to 24 h. Gamma scintigraphy was performed in order to visualize brain uptake following intranasal and intravenous administrations of ^99m^Tc-Lzp-PLGA-NPs. The gamma scintigraphy images were taken in rat after 0.50 h intravenous injection and intranasal administrations of LS and Lzp-PLGA-NPs (Figure 11). The presence of some radioactivity in the esophagus following i.n. administration indicates some percent of drug absorbed into systemic circulation. The scintigraphy images indicate the high uptake of NPs into the brain.

### 3.11. Biodistribution Studies

**Figure 12:**
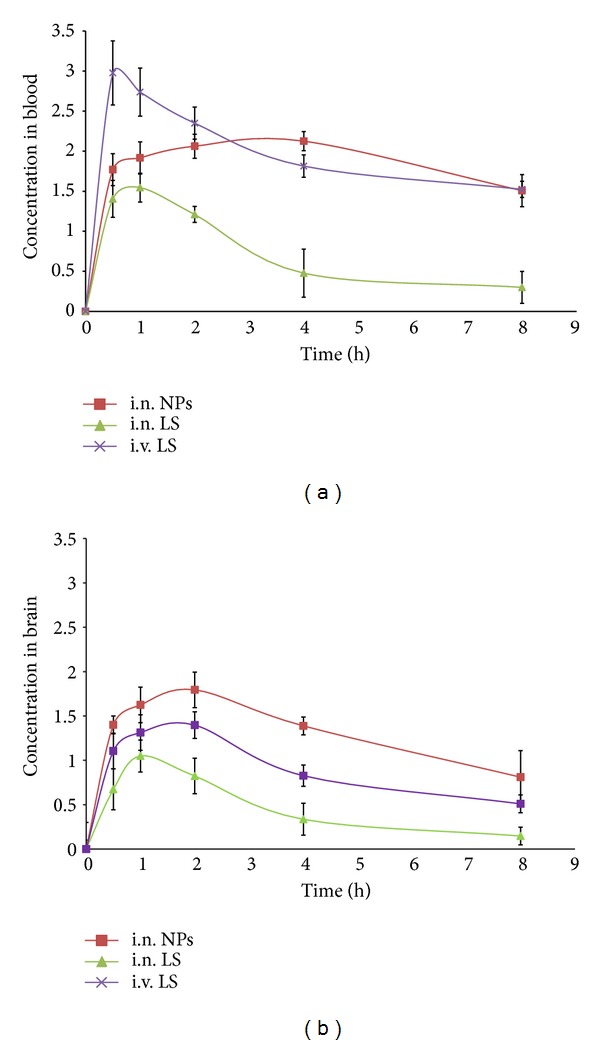
(a) ^99m^Tc-Lorazepam concentration in rat blood at different time intervals following LS (i.v.), LS (i.n.), and Lzp-PLGA-NPs (i.n.) administration. (b) ^99m^Tc-Lorazepam concentration in rat brain at different time intervals following LS (i.v.), LS (i.n.), and lorazepam NPs (i.n.) administration.

Biodistribution studies following i.v. ^99m^Tc-LS, intranasal (i.n.) ^99m^Tc-LS, and i.n. ^99m^Tc- Lzp-PLGA-NPs administration to Sprague-Dawley rats were performed and the radioactivity was estimated at predetermined time intervals up to 8 h. The results obtained are recorded in Table 3 and Figure 12. The brain/blood ratio of the drug at all-time points for different formulations was also calculated and recorded.

The brain/blood ratios of the drug were found to be higher for ^99m^Tc-Lzp-PLGA-NPs when administered intranasally as compared to ^99m^Tc-LS (i.v.) and ^99m^Tc-LS (i.n.). This may be attributed to preferential nose-to-brain transport following nasal administration. The concentrations of the drug in brain following intranasal administration of ^99m^Tc-Lzp-PLGA-NPs were found to be higher at all sampling time points compared to ^99m^Tc-LS (i.v.) and ^99m^Tc-LS (i.n.) up to 8 h after administration.

The substantially higher uptake in the brain with intranasal administration suggests a larger extent of selective transport of lorazepam from nose-to-brain pathway. It was observed that ^99m^Tc-Lzp-PLGA-NPs (i.n.) showed better sustained activity in the brain as compared to i.n. ^99m^Tc-LS. This could be attributed to PLGA matrix.

### 3.12. Accelerated Stability Studies

The result of accelerated stability studies of Lzp-PLGA-NPs is shown in Table 4. No major changes were observed besides a slight increase in *z*-average and a slight decrease in drug content, after storing for three months at accelerated conditions of temperature and humidity.

## 4. Summary and Conclusion

Lorazepam loaded polymeric nanoparticles using PLGA as release controlling polymer showed potential outcome which were optimized using 4-factor, 2-level Box-Behnken design. The dependent responses, that is, percentage drug entrapment and *z*-average, for different combinations of independent variables, that is, polymer, surfactant, w/o phase ratio, and drug concentration, were obtained experimentally and the results were found to fit the quadratic design model. Quantitative effect of independent variables at different levels on the dependent response was investigated by using polynomial equations generated by the model. On the basis of desirable constraints, point predication technique of Box-Behnken design proposed optimized formulation with combination of *X*_1_ 10 mg/mL, *X*_2_ 9.42 mg/mL, *X*_3_ 10, and *X*_4_ 4.5 mg/mL. It can be concluded that Lzp-PLGA-NPs were successfully optimized and developed using Box-Behnken design.

The Lzp-PLGA-NPs showed biphasic release pattern with initial burst release followed by sustained release. Moreover, cytotoxicity study confirms that optimized formulation showed relatively less cytotoxicity than LS. Gamma scintigraphy images showed clear evidence of high uptake of nanoparticles in brain. Biodistribution studies using intranasal route showed higher and sustained brain concentrations for ^99m^Tc-Lzp-PLGA-NPs as compared to ^99m^Tc-LS i.v. and ^99m^Tc-LS i.n. route.

## Figures and Tables

**Table 1:** Independent and dependent variables levels in Box-Behnken design.

	Levels		
	−1	0	1
**Independent variables**			
*X*_1_ = polymer concentration (w/v)	10	35	60
*X*_2_ = surfactant concentration (w/v)	2	8.50	15
*X*_3_ = aqueous/organic phase ratio (v/v)	2	6	10
*X*_4_ = drug concentration (w/v)	1	3	5

**Dependent Variables:**	**Constraints**		
*Y*_1_ = *z*-average (d*·*nm)	Minimize		
*Y*_2_ = % drug entrapment	Maximize		

**Table 2:** Effect of independent process variables on dependent variable.

Formulation	PLGA mg/mL	Poloxamer mg/mL	w/o phase volume ratio	Drug conc. Mg/mL	*z*-Average d*·*nm (±SD)	% Drug entrapment (±SD)	PDI (±SD)
1.	35	2	6	1	211 ± 0.11	70 ± 1.3	0.183 ± 0.002
2.	35	2	6	5	220 ± 0.8	88.48 ± 0.8	0.150 ± 0.003
3.	10	8.50	10	3	176 ± 0.5	83 ± 0.5	0.048 ± 0.001
4.	35	8.50	2	1	177 ± 1.2	71 ± 1.5	0.17 ± 0.004
5.	10	2	6	3	205 ± 0.9	81 ± 0.7	0.315 ± 0.003
6.	10	8.50	6	5	177 ± 1.6	83.5 ± 0.5	0.110 ± 0.002
7.	10	8.50	2	3	184 ± 1.5	75 ± 0.35	0.078 ± 0.002
8.	35	8.50	6	3	197 ± 0.5	86.6 ± 0.65	0.112 ± 0.004
9.	60	8.50	6	1	271 ± 0.8	76 ± 0.22	0.24 ± 0.001
10.	35	15	6	5	192 ± 1.4	84.3 ± 0.35	0.19 ± 0.001
11.	10	15	6	3	177 ± 0.6	80 ± 2.1	0.04 ± 0.003
12.	60	2	6	3	318 ± 1.2	90.1 ± 0.8	0.441 ± 0.002
13.	35	15	10	3	191 ± 1.5	83.5 ± 1	0.17 ± 0.003
14.	60	15	6	3	228 ± 0.5	82 ± 0.4	0.15 ± 0.001
15.	60	8.50	2	3	241 ± 0.4	88 ± 0.85	0.309 ± 0.005
16.	35	15	6	1	182.5 ± 0.5	66.4 ± 0.2	0.09 ± 0.002
17.	35	2	2	3	215 ± 0.7	87.83 ± 0.1	0.15 ± 0.005
18.	60	8.50	6	5	261 ± 0.5	89 ± 1.7	0.20 ± 0.001
19.	35	8.50	2	5	193 ± 1.1	88 ± 1.5	0.10 ± 0.002
20.	10	8.50	6	1	167 ± 0.8	65.5 ± 1.1	0.21 ± 0.005
21.	35	8.50	10	1	192 ± 1.7	69 ± 0.6	0.28 ± 0.001
22.	35	2	10	3	241 ± 1.5	88 ± 1.5	0.21 ± 0.003
23.	35	8.50	10	5	202 ± 1.2	87 ± 1	0.19 ± 0.002
24.	35	15	2	3	186 ± 1.5	84 ± 0.8	0.15 ± 0.002
25.	60	8.50	10	3	283 ± 0.7	88 ± 1.4	0.15 ± 0.006
26.	35	8.50	6	3	193 ± 0.5	85.12 ± 0.7	0.102 ± 0.004

**Table 3:** Distribution of  ^99m^Tc-lorazepam from LS (i.v.), LS (i.n.), and Lzp-PLGA-NPs (i.n.) at different time intervals in Sprague-Dawley rats∗.

Formulation and route of administration	Distribution of lorazepam in blood and brain compartments at different Distribution sampling time points					
	Organ/tissue	0.5 h	1 h	2 h	4 h	8 h
LS (i.v)	Blood	2.976 ± 0.1	2.737 ± 0.15	2.350 ± 0.3	1.813 ± 0.25	1.524 ± 0.15
	Brain	1.547 ± 0.2	1.512 ± 0.3	1.396 ± 0.32	0.826 ± 0.11	0.610 ± 0.12

LS (i.n)	Blood	1.404 ± 0.2	1.545 ± 0.2	1.210 ± 0.04	0.556 ± 0.16	0.359 ± 0.1
	Brain	0.673 ± 0.2	1.148 ± 0.25	0.923 ± 0.2	0.335 ± 0.08	0.247 ± 0.2

Lzp-PLGA-NPs (i.n)	Blood	1.769 ± 0.3	1.916 ± 0.18	2.062 ± 0.18	2.125 ± 0.35	1.507 ± 0.1
	Brain	1.399 ± 0.1	1.624 ± 0.23	1.794 ± 0.15	1.388 ± 0.22	0.104 ± 0.2

LS (i.v)	Brain/blood	0.519 ± 0.23	0.553 ± 0.25	0.594 ± 0.2	0.456 ± 0.05	0.400 ± 0.2

LS (i.n)	Brain/blood	0.479 ± 0.15	0.743 ± 0.1	0.763 ± 0.15	0.602 ± 0.1	0.687 ± 0.3

Lzp-PLGA-NPs (i.n)	Brain/blood	0.791 ± 0.2	0.847 ± 0.15	0.870 ± 0.1	0.653 ± 0.25	0.694 ± 2.5

*The rats were administered 100 *μ*Ci  ^99m^Tc-lorazepam and the radioactivity was measured in percent per gram of tissue of the administered dose. Each value is the mean ± SD of three estimations. Radioactivity was measured at 0 h and all the measurements were performed using 0 h sample corresponding tissue/organ as blank sample.

**Table 4:** Results of stability study conducted on the Lzp-PLGA-NPs for 90 days at 25 ± 2°C and 60 ± 5% RH.

Time (days)	*z*-Average^a^ ± S.D. (d*·*nm)	% Drug remaining
0	168 ± 0.11	100
30	171 ± 0.15	99.78 ± 0.08
60	179 ± 0.085	99.34 ± 0.5
90	186 ± 0.11	98.9 ± 0.3

^a^Not significant (*P* > 0.05).
